# Effect of Normobaric Oxygen Inhalation Intervention on Microcirculatory Blood Flow and Fatigue Elimination of College Students After Exercise

**DOI:** 10.3389/fgene.2022.901862

**Published:** 2022-06-01

**Authors:** Yong Peng, Liang Meng, Huan Zhu, Li Wan, Fen Chen

**Affiliations:** ^1^ School of Physical Education, Hubei Minzu University, Enshi, China; ^2^ Graduate Schools, Moscow State Academy of Physical Culture, Malakhovka, Russia; ^3^ Sports Department, Suzhou University of Science and Technology, Suzhou, China

**Keywords:** atmospheric oxygen inhalation, microcirculatory blood flow, muscle oxygen saturation, exercise-induced fatigue, fatigue elimination

## Abstract

**Objective:** To explore the effect of normobaric oxygen inhalation intervention on microcirculatory blood flow of college students after exercise and the impact of the elimination of exercise-induced fatigue, to provide a theoretical and methodological reference for the rapid elimination of fatigue of college students after endurance exercise.

**Methods:** Forty-eight male non-sports majors of Hubei University for nationalities were randomly divided into the control group (n = 24) and intervention group (n = 24). The subjects in both groups completed the same exercise program twice (running 3,000 m on the treadmill at maximum speed). After running, the issues in the intervention group inhaled portable oxygen for 30 min, and the control group recovered naturally. Microcirculatory blood flow (MBP), blood flow velocity (AVBC), blood flow concentration (CMBC), muscle oxygen saturation (SmO2), heart rate (HR), blood lactic acid (BLA), blood urea (BU), and creatine kinase (CK) were measured before exercise, immediately after exercise and 30 min after exercise.

**Results:** 1) MBP and AVBC had interaction between groups and time before and after exercise, MBP and AVBC immediately after exercise in the intervention group were significantly higher than those before exercise and 30 min after exercise, and 30 min after exercise in the intervention group were significantly higher than those in the control group. 2) SmO2, HR, BLA, BU, and CK had interaction between groups and time, and SmO2 immediately after exercise in the intervention group was significantly lower than that before exercise and 30 min after exercise, but significantly higher than that in the control group at 30 min after exercise. The HR and BLA immediately after exercise in the intervention group were significantly higher than those before exercise and 30 min after exercise, but significantly lower than those in the control group at 30 min after exercise, and the BU and CK in the intervention group were significantly higher than those before exercise, but significantly lower than those in the control group at 30 min after exercise.

**Conclusion:** Normobaric oxygen inhalation for 30 min after exercise can delay the decrease of microcirculatory blood flow, increase muscle oxygen saturation, and promote the recovery of heart rate, blood lactic acid, blood urea and creatine kinase. Therefore, normobaric oxygen inhalation for 30 min after exercise can be used as an effective means to promote the elimination of exercise-induced fatigue after endurance running.

## 1 Introduction

In recent years, with the continuous decline of college students’ physical health, the probability of adverse cardiovascular events after exercise has gradually increased. The fatigued state of high stress after high intensity exercise is the main factor leading to sports injury and damaging cardiovascular disease of college students, especially when students’ physique is poor, high intensity exercise brings more significant risks. Therefore, the rapid recovery of physical function and the immediate elimination of fatigue after high intensity exercise is of great significance to reduce the occurrence of adverse sports injuries of college students. Atmospheric oxygen inhalation, as an effective method to promote the elimination of fatigue after exercise, is not only practical, but also convenient and straightforward to operate, and has been widely used in competitive sports. Yang Yantao’s study found that rapid oxygen inhalation after competition can significantly reduce the levels of serum creatine kinase and lactate dehydrogenase and increase the content of immunoglobulin (Yang et al., 2016). Oxygen inhalation before and after the match can promote the clearance of lactic acid after the match, improve the ability of antioxidation and accelerate the elimination of exercise-induced fatigue (Xie et al., 2016). Other studies have shown that oxygen inhalation in middle-and long-distance running can reduce students’ cardiovascular stress levels, which has a positive effect on students’ psychology (Zhang, 2019). Although oxygen inhalation contributes to the elimination of fatigue after exercise, the timely elimination of metabolic waste after exercise and the rapid transport of energy and oxygen to skeletal muscle and other organs are essential factors that determine the effect of fatigue elimination. Microcirculation is the only place for material and energy exchange, and its blood perfusion level is an essential condition to complete this process. The previous study of the author’s team shows a close relationship between microcirculation blood flow and the occurrence of exercise-induced fatigue, and increasing microcirculation blood flow is helpful to reduce the occurrence of fatigue (Zhu and Binghong, 2016; Zhang et al., 2017; Zhu and Gao, 2019). However, at present, there are few studies on the effect of oxygen inhalation on microcirculation blood flow after exercise, and it is not clear whether oxygen inhalation can improve microcirculation blood flow after exercise. Based on this, through the intervention of rapid oxygen inhalation of college students after 3000-m running, this study discusses the effect of oxygen inhalation on microcirculatory blood flow and fatigue elimination after exercise, to provide a theoretical and methodological reference for the rapid elimination of fatigue after endurance exercise.

## 2 Research Objects and Methods

### 2.1 Research Object

Forty-eight non-sports male students of Hubei University for nationalities were selected as the research subjects, which met the following criteria: 1) after the unified physical examination of the research group (the physical examination center of the affiliated Hospital of Hubei University for nationalities), there were no cardiovascular diseases such as hypertension and coronary heart disease, respiratory diseases, chronic kidney disease, and liver disease; 2) BMI <24; 3) no history of operation, a limb movement disorder. 4) exercise at least 1–2 times a week recently, and be able to bear a certain exercise load; 5) voluntarily participate in and sign a written consent form after being informed of the test process and purpose. Forty-eight subjects were randomly divided into control group (n = 24) and intervention group (n = 24), The ratio of male to female in the experimental group and the control group was the same, and there was no gender difference. This study was approved by the Biomedical Ethics Committee of Hubei University for nationalities (approval document No.: HBMZDX2022042). The basic conditions of the two groups are shown in Table 1 below.

**TABLE 1:** Basic information of subjects (x ± s, n = 48).

Group	Age (years)	Height (m)	Weight (kg)	BMI (kg/M^2^)
Control group	21.07 ± 0.59	1.73 ± 0.05	66.80 ± 4.89	22.18 ± 1.02
Intervention group	21.41 ± 0.63	1.72 ± 0.06	65.40 ± 6.39	22.09 ± 1.27

### 2.2 Test Method Design

**FIGURE 1:**
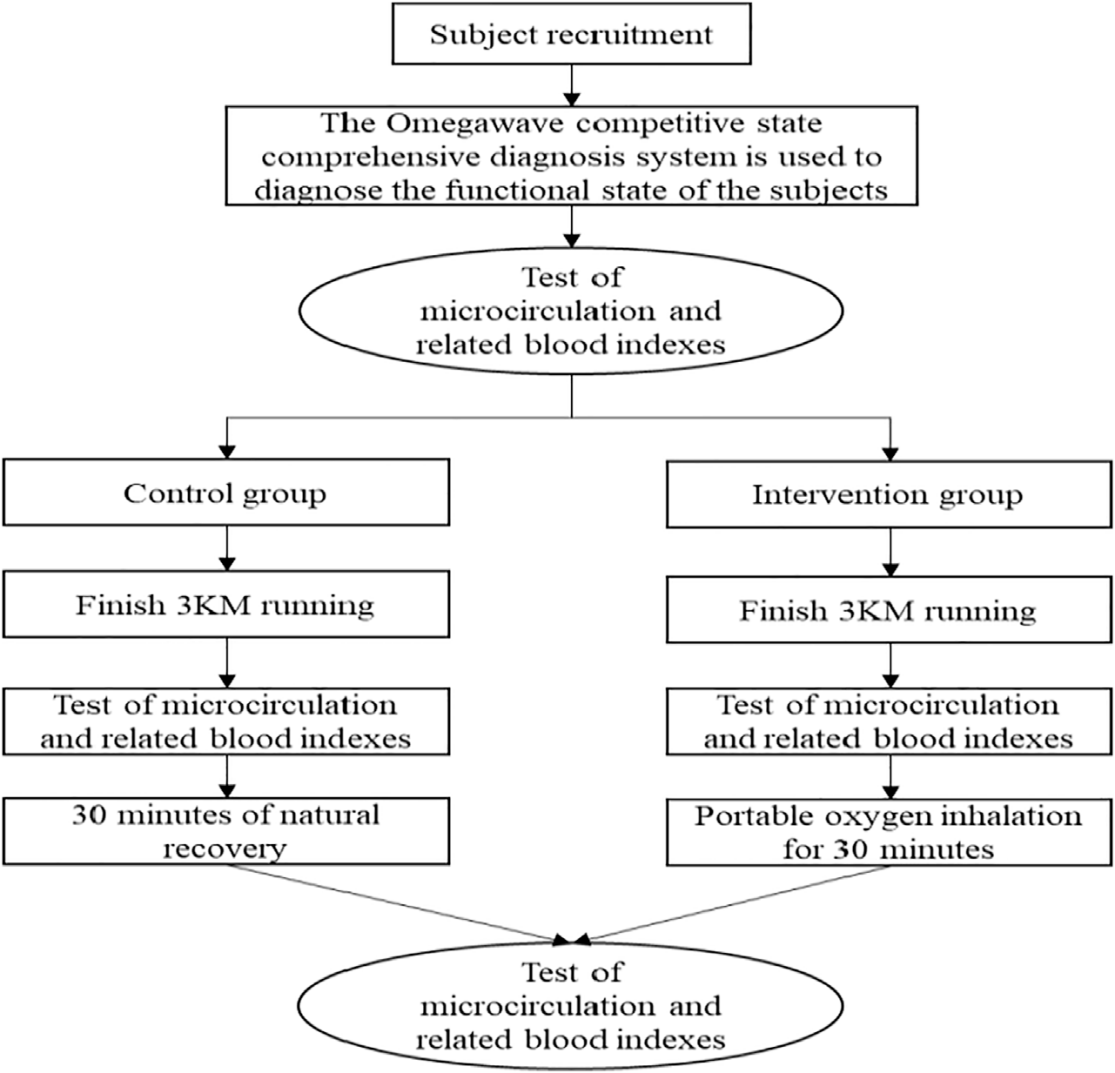
The test flow chart of this study.

The subjects sat in the lab 30 min earlier and wore a team heart rate meter. After sitting for 30 min, the subjects’ microcirculatory blood flow, muscle oxygen saturation, heart rate, blood lactic acid, blood urea, creatine kinase (venous blood drawing), and other indexes were collected. The issues then ran 3,000 m (the subjects ran 3,000 m at the fastest speed on the treadmill). After the end of running, the above-mentioned indexes of the subjects were collected again. After the end of index collection, the issues in the intervention group inhaled portable oxygen for 30 min, the control group recovered naturally; after the end of 30 min, the above-mentioned indexes were collected again. Before the experiment, immediately after the experiment and 30 min after the experiment, the collection of microcirculation index and blood index were carried out at the same time, and there was no time difference. To ensure the stability of the operating condition of the issues, the American Omegawave competitive state comprehensive diagnosis system was used to diagnose the functional state of the subjects before exercise to ensure that the operating conditions of the two groups were the same before exercise. Environment is an important factor affecting vascular blood flow, so this study strictly controls the subjects’ ambient temperature, humidity and indoor air flow in the test process to ensure the consistency of the test environment. The specific test process is shown in Figure 1.

### 2.3 Oxygen Suction Equipment

In this study, a portable oxygen-making machine made by Shandong Jing’an Medical Devices Co.Ltd., was used (model: P2MurW). The oxygen concentration was (90–96)%, the flow rate was 1L/min, and the oxygen discharge mode was pulse-type (equivalent to ordinary 5L/min oxygen machine effect). The oxygen supply of the instrument is stable and can meet the oxygen supply-demand when the respiratory rate is between 10 and 40 beats per minute.

### 2.4 Test Index and Instrument

#### 2.4.1 Microcirculatory Blood flow、Muscle Oxygen Saturation

PF6010 dual-channel laser Doppler blood flow detector made in Sweden was used to measure the blood flow of the skin of the middle muscle of the quadriceps femoris of the right lower limb of the subjects. The test indexes included mean movement velocity of blood cells (AVBC), exercise blood cell concentration (CMBC), and microvascular blood perfusion (MBP), including MBP = AVBC*CMBC/100. The instrument for measuring muscle oxygen saturation (SmO2) is Moxy near-infrared wireless muscle oxygen testing system, and the test site is the same as microcirculatory blood flow.

#### 2.4.2 Heart rate、Blood Lactic acid、Blood urea、Creatine Kinase

The heart rate (HR) acquisition instrument is the heart rate meter of the Polar team made in Finland. Blood lactic acid (BLA) was collected by German EKFLactateScout4 portable blood lactic acid analyzer. Blood urea (BU) and creatine kinase (CK) were tested in the laboratory. After venous blood was drawn, the blood was centrifuged, and then the serum was stored in a refrigerator of minus 80°, and then sent to the hospital for testing.

### 2.5 Statistical Method

Use SPSS25.0 to count the data. The normal distribution of the data was tested. The time and energy consumption of the subjects running 3,000 m before and after running twice were tested by independent sample *t*-test, and the other indexes were compared by the analysis of repeated measurement variance.

## 3 Result

### 3.1 The Time and Energy Consumption of the Two Groups of Subjects Running 3,000 m

There was no significant difference in the time and calories consumed in running 3,000 m between the intervention group and the control group, as shown in Table 2.

**TABLE 2:** Comparison of time and energy consumption of two groups of subjects in running 3,000 m (x ± s, n = 48).

Group	Exercise time (s)	Energy consumption (kcal)
Control group	997.13 ± 81.55	186.87 ± 5.77
Intervention group	975.33 ± 84.06	192.25 ± 6.53

### 3.2 Comparison of Microcirculation Perfusion in Different Periods Between Two Groups of Subjects

Table 3 showed that MBP and AVBC had interaction between groups and time before and after the test. Simple effect analysis showed that the group had different effects on MBP and AVBC, the MBP and AVBC immediately after exercise in the control group were significantly higher than those before exercise and 30 min after exercise, and the MBP and AVBC immediately after exercise in the intervention group were significantly higher than those before exercise and 30 min after exercise, and the MBP and AVBC before exercise were substantially lower than those at 30 min after exercise. Time had a different effect on MBP and AVBC. In the intervention group, MBP and AVBC before exercise were significantly lower than 30 min after exercise (*p* < 0.05). Before and after the experiment, CMBC had no interaction between group and time (*p* > 0.05).

**TABLE 3:** Comparison of microcirculation perfusion in different periods between two groups of subjects (x ± s, n = 48).

Index	Group	Before exercise	Immediately after exercise	30 min after exercise	Interaction	
					F	P
MBP(PU)	Control group	6.05 ± 1.86*	37.25 ± 8.51	6.34 ± 2.14*^&^	4.066	0.028
	Intervention group	6.19 ± 1.91*^#^	39.83 ± 5.92	10.13 ± 2.16*		
CMBC	Control group	62.08 ± 18.18	214.80 ± 66.12	65.57 ± 13.82	0.032	0.969
	Intervention group	64.57 ± 19.82	220.93 ± 62.06	68.07 ± 9.09		
AVBC	Control group	9.93 ± 2.60*	18.91 ± 7.32	10.23 ± 2.38*^&^	3.959	0.031
	Intervention group	9.79 ± 2.03*^#^	19.81 ± 6.06	15.49 ± 4.24*		

Note: * the difference is statistically significant compared with the corresponding index immediately after exercise in the group; # the difference between the corresponding index in the group and 30 min after exercise in the group is statistically significant; & the difference is statistically significant compared with the index at the same time in the group.

### 3.3 Comparison of SmO2, HR, BLA, BU, and CK Between Two Groups of Subjects in Different Periods

Table 4 showed that SmO2, HR, BLA, BU, and CK had interaction between groups before and after the test. Simple effect analysis showed that the group had different effects on SmO2, HR, BLA, BU, and CK. In the control group, SmO2 immediately after exercise was substantially lower than that before exercise and 30 min after exercise. At the same time, HR, BLA, BU, and CK immediately after exercise were substantially higher than those before exercise. HR and BLA immediately after exercise were substantially higher than those at 30 min after exercise. SmO2 before exercise was substantially higher than at 30 min after exercise, while HR, BLA, BU, and CK before exercise were substantially lower than those at 30 min after exercise. In the intervention group, SmO2 immediately after exercise was substantially lower than before exercise and 30 min after exercise. At the same time, HR, BLA, BU, and CK immediately after exercise were substantially higher than those before exercise and 30 min after exercise. BLA, BU, and CK before exercise were substantially lower than those at 30 min after exercise. Time had separate effects on SmO2, HR, BLA, BU, and CK. 30 min after exercise, SmO2 in the control group was substantially lower than that in the intervention group. In contrast, HR, BLA, BU, and CK in the control group were substantially higher than those in the intervention group.

**TABLE 4:** Comparison of SmO2, HR, BLA, BU and CK between two groups of subjects in different periods (x ± s, n = 48).

Index	Group	Before exercise	Immediately after exercise	30 min after exercise	Interaction	
					F	P
SmO2	Control group	67.91 ± 9.61*^#^	48.24 ± 12.00	56.89 ± 9.32*^&^	3.987	0.025
	Intervention group	68.32 ± 9.44*	46.04 ± 9.98	64.15 ± 8.87*		
HR	Control group	76.87 ± 8.03*^#^	174.20 ± 11.53	91.40 ± 7.23*^&^	3.693	0.038
	Intervention group	77.13 ± 7.37*	175.47 ± 5.94	82.40 ± 7.47*		
BLA	Control group	2.99 ± 0.50*^#^	15.87 ± 2.54	6.08 ± 1.66*^&^	4.771	0.017
	Intervention group	2.88 ± 0.42*^#^	16.14 ± 2.44	4.45 ± 1.07*		
BU	Control group	4.23 ± 0.59*^#^	6.20 ± 0.89	6.22 ± 0.85^&^	5.244	0.025
	Intervention group	4.28 ± 0.51*^#^	6.46 ± 1.06	5.48 ± 0.87*		
CK	Control group	122.27 ± 28.02*^#^	324.60 ± 49.22	342.47 ± 64.38^&^	11.147	0.000
	Intervention group	125.67 ± 27.07*^#^	325.73 ± 49.06	270.27 ± 51.29*		

Note: * the difference is statistically significant compared with the corresponding index immediately after exercise in the group; # the difference between the corresponding index in the group and 30 min after exercise in the group is statistically significant; & the difference is statistically significant compared with the index at the same time in the group.

## 4 Discuss

After high intensity exercise, metabolic wastes such as lactic acid, carbon dioxide, and adenosine can be eliminated in time, and energy substances (oxygen, glucose, etc.) can be quickly transported to cells, thus promoting the rapid elimination exercise-induced fatigue and the quick recovery of body function. As the only place of material and energy metabolism, microcirculation is the key to completing this process. Insufficient microcirculatory blood perfusion after exercise will not only slow down the elimination of metabolic waste after exercise, but also cause the exchange of energy materials, resulting in the accumulation of metabolic waste in cells, thus unable to obtain sufficient energy materials and oxygen in time. In addition, the lack of microcirculation blood perfusion will also lead to the disturbance of material and energy metabolism during exercise, which will lead to insufficient oxygen supply during exercise, induce exercise-induced fatigue and reduce exercise performance. Therefore, the amount of microcirculatory blood perfusion is closely related to the recovery of physical function and exercise-induced fatigue of athletes after exercise. Increasing the amount of microcirculatory blood perfusion is helpful to improve the exchange capacity of oxygen and nutrients between blood and tissues, increase the efficiency of systemic blood circulation, and promote the rapid recovery of the body after exercise and the elimination of fatigue.

In this study, the microcirculatory blood flow of the two groups increased significantly after the end of 3000-m running. The increase of microcirculation blood flow during exercise is mainly related to the release of endogenous NO. In the process of exercise, the blood flow in the blood vessel increases, which increases the fluid shear stress of the blood to the blood vessel wall, and the shear stress can promote the vascular endothelial cells to produce endogenous NO, promote vasodilation and increase blood flow (Wang and Cai, 2012; Zhu and Gao, 2020). In addition, the production of hydrogen ions, adenosine, and other metabolites during exercise can also stimulate vascular endothelial cells and improve vasodilation ability and blood perfusion level (Binggeli et al., 2003; Cracowski et al., 2007; Lorenzo and Minson, 2007). The increase of microcirculation blood flow during exercise can provide energy and oxygen for skeletal muscle and other organs to meet the metabolic needs of the body; at the same time, the metabolic waste produced during exercise can be removed from the body in time. After the exercise, due to the excessive oxygen consumption, the subjects are still in high intensity operation, and metabolic wastes such as lactic acid continue to be produced. Therefore, keeping the microcirculation blood flow at a certain level after exercise is helpful to the elimination of metabolic waste and the transport of energy and oxygen, accelerating the elimination of fatigue and promoting the recovery of cell function. In this study, the microcirculation blood flow of the subjects in the intervention group was significantly higher than that in the control group after 30 min of portable oxygen inhalation, indicating that inhaling high concentration oxygen after exercise could slow down the decline of microcirculation blood flow. The effect of oxygen inhalation on microcirculation blood flow may be related to the release of NO. Studies have shown that oxygen inhalation during exercise can significantly increase the levels of eNOS and NO in blood after exercise, and increase the level of blood perfusion after exercise (Cui et al., 2005). In addition, the effect of oxygen inhalation on microcirculation blood flow after exercise may be related to the deformability of red blood cells. After strenuous exercise, many intracellular Ca2+ gathered and activated the K+ ion channel on the erythrocyte membrane, which led to the deformation of red blood cells. Inhaling a high oxygen concentration after exercise could promote the morphological changes of red blood cells to be double flattened, increase the contact area with oxygen and improve the movement speed of blood cells (Xiao et al., 2002).

Muscle oxygen saturation mainly refers to the dynamic balance of oxygen supply and oxygen consumption in arteries, veins, and capillaries of skeletal muscle and the overall effect of myoglobin content, equal to (oxygenated hemoglobin + oxygenated myoglobin)/(hemoglobin + myoglobin). After oxygen enters the blood, it combines with hemoglobin to form oxygenated hemoglobin, which is then transported to cells (especially muscle cells) with the help of capillaries to be used by cells. At the same time, myoglobin in muscle will also combine part of oxygen to be stored for cell activity to form oxygenated myoglobin. The combination of oxygen and myoglobin directly affects the ability of myoglobin to store oxygen. Under normal circumstances, the blood oxygen saturation can reach more than 98%.Still, the muscle oxygen saturation (the comprehensive reaction of hemoglobin and myoglobin to bind oxygen) is lower, indicating that under normal circumstances, the ability of myoglobin oxygenation is much less than that of hemoglobin oxygenation. Myoglobin oxygenation ability mainly depends on the ability of capillaries to transport oxygen to muscle cells. In this oxygen, partial pressure difference is the power to promote oxygen from veins to muscle cells. The greater the oxygen partial pressure of microvessels, the greater the pressure difference between microvessels and muscle cells, the greater the rate of oxygen from capillaries into muscle cells, the higher the content of muscle, and the stronger the oxygenation ability of myoglobin. The higher the partial pressure of oxygen, the greater the pressure difference between the oxygen and muscle cells, which accelerates the rate of oxygen from capillaries to muscle cells, so the oxygen content in muscle increases and the oxygenation ability of myoglobin increases. Therefore, compared with blood oxygen saturation, muscle oxygen saturation can better reflect the functional recovery of skeletal muscle cells after exercise.

In the exercise process, due to the massive consumption of oxygen, the muscle oxygen saturation decreased significantly. After exercise, with the establishment of oxygen supply-aerobic balance, muscle oxygen saturation gradually recovers, so muscle oxygen saturation can objectively reflect the change of oxygen supply capacity of the body after exercise. In this study, the muscle oxygen saturation of the subjects in the intervention group was significantly higher than that in the control group after portable oxygen inhalation for 30 min, indicating that oxygen inhalation after exercise can improve the oxygen supply capacity of the body and promote the recovery of muscle oxygen saturation, which is similar to the research results of relevant scholars. Studies have shown that oxygen supplementation during exercise can significantly prolong the endurance time and the lowest muscle oxygen saturation of mice (Marillier et al., 2021). The intervention effect of oxygen inhalation on muscle oxygen saturation is closely related to the change of microcirculatory blood flow. As mentioned earlier, the oxygen partial pressure difference between capillaries and muscle cells is the driving force to increase the oxygen diffusion rate and distance. The microcirculation blood flow is the carrier to carry and transport oxygen: the greater the microcirculation blood flow, the greater the capillary oxygen partial pressure. Therefore, the oxygen partisan pressure difference between capillaries and muscle cells will also increase.

Heart rate is the most direct and straightforward index to reflect the exercise intensity and exercise recovery of the human body. It is found that inhaling high oxygen for 30 min after exercise can accelerate the recovery speed of heart rate and lactic acid (Tao, 2011). Other studies have shown that although oxygen inhalation during exercise has no significant effect on the oxygen consumption of rowers, oxygen inhalation after exercise can significantly reduce the level of blood lactic acid and heart rate of rowers before the next exercise (Pelvan and Çotuk, 2016). This study found that the heart rate of the subjects increased significantly after 3000-m running, but inhaling hyperoxia for 30 min immediately after exercise could accelerate the recovery of heart rate, which was related to the change of sympathetic nerve function. Oxygen inhalation after exercise can enhance the function of the vagus nerve and heart rate variability, quickly recover heart rate, and reduce cardiac oxygen consumption (Xie, 2016). The content of blood lactic acid in the human body is low in a quiet state, and a large amount of lactic acid will be produced after high intensity exercise. The lack of timely decomposition or oxidation of lactic acid in the body will lead to the decline of cell function (inhibiting the activity of enzymes) and induce exercise-induced fatigue. It is found that the elimination of lactic acid can be accelerated by inhaling atmospheric pressure and high concentration of oxygen after a 400-m race (Xie et al., 2016). In addition, studies have found that inhaling hyperoxia after exercise can increase oxygen content in the blood, alleviate the blood oxygen supply after strenuous exercise, accelerate the speed of intracellular H+ movement, and promote the elimination of lactic acid (Tao, 2011). In addition, studies have shown that oxygen inhalation during exercise can improve endurance exercise performance and reduce the concentration of lactic acid after exercise (Silva et al., 2021). In this study, the concentration of blood lactic acid increased significantly after 3000-m running. Still, the clearance of lactic acid was accelerated by inhaling high oxygen for 30 min immediately in the intervention group. The main pathways of lactic acid after exercise are direct oxidative decomposition, gluconeogenesis for saccharification storage, synthesis of fatty acids and other substances, and directly removed from the body with urine and body fluids, etc.,Still, all ways must be completed with the help of blood circulation. Therefore, increasing the blood flow of microcirculation after exercise can accelerate the oxidative decomposition of lactic acid and promote the recovery of cell function.

Blood urea is the product of protein decomposition and is an important index to evaluate exercise-induced fatigue. Blood urea level is mainly affected by renal function, protein intake, and catabolism. When athletes are in a state of exhaustion, the energy supply of fat oxidation decreases, the proportion of protein-energy supply increases, and the decomposition rate increases, increasing blood urea content. Creatine kinase is a standard index to evaluate exercise intensity. Under the stimulation of hypoxia, mechanical stress, and traction, the permeability of skeletal muscle cell membrane will change, resulting in the flow of creatine kinase from cells to the blood, resulting in an increase in the content of creatine kinase in the blood. The results showed that after 30-s maximum power cycling and exercise-induced fatigue in rats, the renal function was restored, and the blood urea level decreased significantly (Teng et al., 2013; Xu, 2014; Guoyin, 2019). In addition, other studies have shown that a high concentration of oxygen inhalation after high intensity exercise can reduce the contents of creatine kinase, creatine kinase isoenzyme, and lactate dehydrogenase, and prevent the occurrence of sports injury (Xie et al., 2016; Yang et al., 2016). In this study, after 30 min of high concentration oxygen supplementation, the levels of blood urea and creatine kinase in the intervention group decreased significantly, indicating that high concentration oxygen supplementation after high intensity exercise can promote the recovery of cellular functions such as liver and skeletal muscle, and reduce the occurrence of exercise-induced fatigue. In the process of high intensity exercise, renal hypoxia leads to the decrease of glomerular filtration ability, resulting in the accumulation of blood urea in the body. At the same time, oxygen inhalation intervention improves the occurrence of renal hypoxia by increasing the blood flow of renal tissue, so that the renal function can be recovered. The urea accumulated in the blood is removed. In addition, a high concentration of oxygen supplementation after exercise can also promote the aerobic metabolism of fat and sugars, reduce the decomposition of protein, and reduce the content of urea in the blood. Therefore, the intervention of high concentration oxygen supplementation after exercise-induced fatigue can promote the recovery of blood urea levels. In addition, oxygen supplementation after exercise can promote the healing of skeletal muscle cell membrane permeability caused by hypoxia and make skeletal muscle cells maintain normal function. However, some studies have shown that the change of creatine kinase level after high intensity exercise mainly reflects delayed muscle soreness. High concentration oxygen supplementation can not improve muscle soreness and creatine kinase level (Shelina et al., 2003; Bennett, 2014; Navid et al., 2020). The authors believe that the effect of a high concentration of oxygen supplementation on creatine kinase after high intensity exercise is related to the changes in muscle cell membrane permeability. When the muscle fiber structure is physiologically damaged by eccentric exercise, the intervention effect of high concentration oxygen supplementation may not be good. Still, when hypoxia leads to the increase of muscle membrane permeability, high concentration oxygen supplementation can promote the recovery of cell function by improving the level of skeletal muscle blood perfusion and oxygen supply capacity. In this study, the subjects took the 3000-m race as the exercise content (without centrifugal exercise). Hypoxia caused changes in cell membrane permeability after exercise, while a high concentration of oxygen supplementation promoted the recovery of cell membrane permeability and decreased the level of creatine kinase.

## 5 Conclusion

Normobaric oxygen inhalation for 30 min after exercise can delay the decrease of microcirculatory blood flow, increase muscle oxygen saturation, and promote the recovery of heart rate, blood lactic acid, blood urea, and creatine kinase. Therefore, normobaric oxygen inhalation for 30 min after exercise can be used as an effective means to promote the elimination of exercise-induced fatigue after endurance running.

## Data Availability

The original contributions presented in the study are included in the article/supplementary material, further inquiries can be directed to the corresponding author.
